# Hyperferritinemia as a Clue to Neuroendocrine Carcinoma

**DOI:** 10.7759/cureus.90020

**Published:** 2025-08-13

**Authors:** Eugene S Chao, Gene Yoshikawa, Xin Qing, Charity Huang

**Affiliations:** 1 Internal Medicine, Harbor University of California Los Angeles Medical Center, Torrance, USA; 2 Hematology Oncology, Harbor University of California Los Angeles Medical Center, Torrance, USA; 3 Pathology, Harbor University of California Los Angeles Medical Center, Torrance, USA

**Keywords:** anemia workup, hyperferritinemia, neuroendocrine carcinoma of prostate, neuroendocrine differentiation, poorly differentiated metastatic carcinoma

## Abstract

Ferritin is the protein that serves as the main mechanism for iron storage. It can also be an acute-phase reactant, which may rise in response to inflammation, infection, injury, autoimmune disease, or malignancy. Therefore, when evaluating ferritin levels, it is important to consider both iron storage and potential underlying conditions that could cause hyperferritinemia. We report a case of an elderly man in his late 80s who had elevated ferritin levels for years before ultimately being diagnosed with metastatic neuroendocrine carcinoma. The initial diagnosis was suspected to be due to iron overload from iatrogenic causes, but despite discontinuation of iron supplementation, the patient continued to have hyperferritinemia. Broad differential diagnoses were considered, including hereditary hemochromatosis, sideroblastic anemia, thalassemia, viral infection, hereditary hyperferritinemia, myelodysplastic syndromes with ineffective iron regulation, hemophagocytic lymphohistiocytosis, and other autoimmune phenomena. However, workup was negative. Ultimately, bone marrow biopsy was performed, which revealed poorly differentiated metastatic carcinoma with neuroendocrine differentiation.

## Introduction

Ferritin is the protein responsible for iron storage, forming multimeric assemblies with crystalline iron cores [1]. Clinically, abnormal ferritin often reflects disturbances in iron homeostasis or metabolism. Elevated ferritin can be observed in iron-overloaded states such as hereditary hemochromatosis and transfusion iron overload [2]. Ferritin is also an acute-phase reactant, rising in response to inflammation, bacterial and viral infection [3], injury, autoimmune disease, and malignancy [4]. Due to the extensive range of associated conditions, the differential diagnosis should remain broad, and further workup should be performed. 

Prior studies have observed a correlation between increased ferritin concentrations and diverse malignant conditions. As early as the 1980s, a study of 21,513 Chinese male government workers in Taiwan showed that the relative risk of all cancer death for men with a serum ferritin level of 200ng/mL was 2.9 compared to men with a serum ferritin level of 20ng/mL [5]. Later studies have shown that various malignancies, including neuroendocrine neoplasms, are associated with elevated ferritin levels [4]. The light chain of the ferritin molecule has been known to be upregulated under hypoxic conditions, which are commonly observed in malignancy. Specifically, the direct binding of hypoxia-inducible factor 1-alpha (HIF 1α) to the hypoxia response element (HRE-3) region of the ferritin light chain promoter has been implicated in increased transcription activity of ferritin in the setting of tumor growth and metastasis [6].

Neuroendocrine neoplasms are a heterogeneous group of malignancies with cells containing both “neuro” and “endocrine” properties [7]. The “neuro” property derives from the dense-core granules also present in serotonergic neurons [8], whereas the “endocrine” property derives from the synthesis and secretion of monoamines [9]. Neuroendocrine tumors can arise from various endocrine glands, including the pituitary, parathyroid, adrenals, the islets of thyroid and pancreas, and from cells lying between exocrine glands such as gastroenteropancreatic and respiratory tracts. There are approximately 100,000 cases of neuroendocrine tumors in the US [9], making it a somewhat rare diagnosis. Here, we present a challenging diagnostic case of a patient with elevated ferritin levels for years, where broad differential diagnoses were considered, ultimately leading to an unexpected diagnosis of metastatic neuroendocrine carcinoma (NEC).

## Case presentation

We present a case of an elderly man in his late 80s with a past medical history notable for hypertension, hypothyroidism, benign prostatic hyperplasia, bilateral cataracts, and hearing loss, who was referred to hematology for suspected iron overload.

Starting in 2017, and possibly before that, the patient was noted to have macrocytic anemia with hemoglobin (Hgb) levels between 12 and 13 (reference range: 13.5-16.5 g/dL) and a mean corpuscular volume (MCV) in the low 100s (reference range: 82.0-97.0 fL). Despite these findings, the macrocytic anemia had been stable, and the patient remained asymptomatic. Reportedly, the patient had undergone a colonoscopy in the past without any apparent abnormalities; however, he had no documentation of the findings. Given persistent anemia, oral iron supplementation was empirically initiated by the patient’s primary care provider in mid-2020. 

In mid-2021, the ferritin level was found to be elevated to 1718. By early 2022, iron saturation was elevated to 67%, and total iron-binding capacity (TIBC) was low at 240 (reference range: 261-478 mcg/dL). These findings raised concerns for iron overload. Vitamin B12, folate, and TSH levels were all within normal limits. On initial hematology evaluation, the patient endorsed generalized fatigue for the past year without other symptoms such as bruising, bleeding, abdominal pain, headaches, or tinnitus. The physical exam was grossly benign. It was thought that the patient’s hyperferritinemia was likely due to ferrous sulfate administration. The patient was advised to discontinue ferrous sulfate tablets and reduce wine consumption, as it was thought this could have contributed to macrocytosis. Initially, ferritin levels trended down to 1420 but subsequently rose to 1595 and then to 1824 by early 2024 despite discontinuation of ferrous sulfate. Iron saturation also trended down but then rose to 88% by early 2024. During that period, he had stable macrocytic anemia, with Hgb in the 11 to 13 range and MCV in the 101 to 103 range. Throughout this period, the patient remained asymptomatic, except for stable generalized fatigue, with continued physical exam findings.

**Figure 1:**
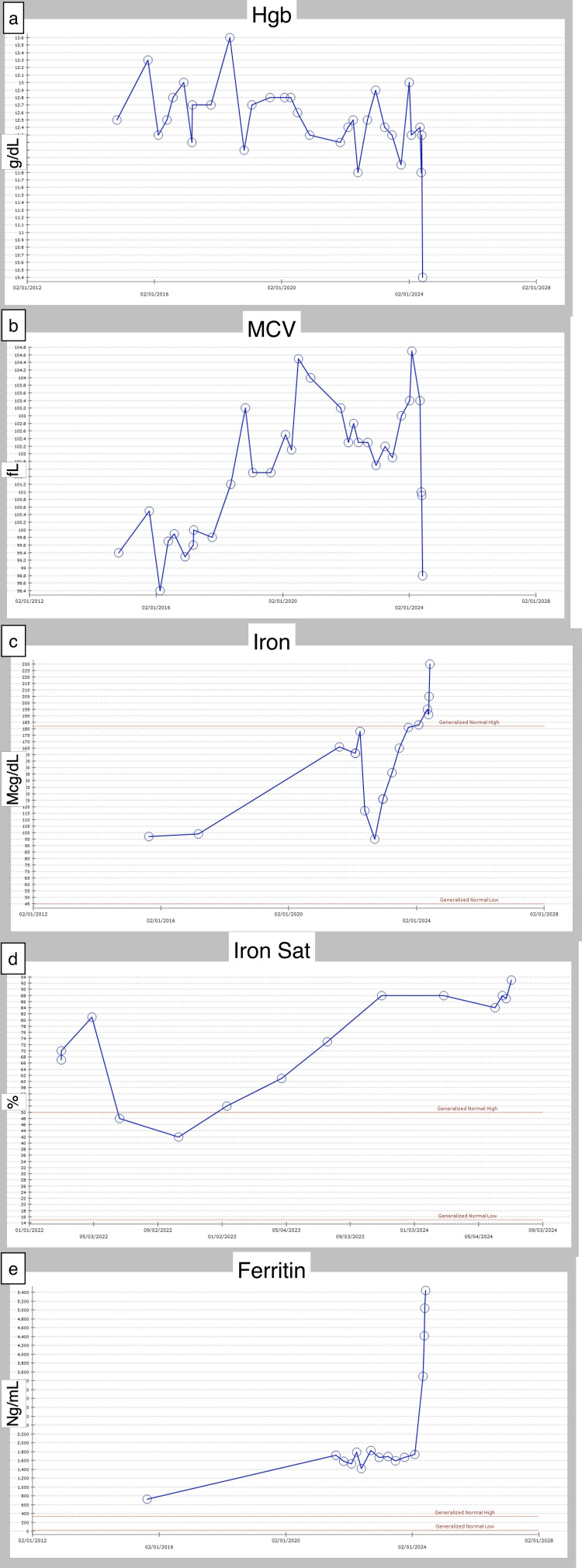
Timeline of Patient’s Hemoglobin, MCV, Iron, Iron Saturation, and Ferritin Levels As shown in (e), ferritin levels continued to rise, from 1,718 ng/mL in mid-2021 to greater than 5,000 ng/mL by early 2024, despite cessation of iron supplementation in October 2022. (a) Hemoglobin level. (b) MCV. (c) Iron level. (d) Iron saturation. (e) Ferritin level MCV: Mean corpuscular volume

Given the persistently elevated ferritin and iron levels despite stopping iron supplementation, hereditary hemochromatosis was considered. Tests for hemochromatosis (HFE) mutation, including C282Y and H63D, were ordered, yielding negative results. However, an abdominal MRI performed in mid-2024 did suggest hepatic iron deposition. Other possible diagnoses, such as sideroblastic anemia, thalassemia (thought to be less likely given low RBC count and macrocytosis), viral infection, hereditary hyperferritinemia, myelodysplastic syndromes (MDS) with ineffective iron regulation, hemophagocytic lymphohistiocytosis (HLH), and other autoimmune phenomena, were considered but seemed less likely (Table 1). Typically, iron overload is a result of anemia of chronic disease [10] or hemolytic anemia [11]. Therefore, patient’s macrocytic anemia was thought to be unrelated to iron overload. Given concern for a possible primary bone marrow process, bone marrow biopsy was recommended in late 2023, but the patient declined. Patient’s ferritin continued to up-trend, rapidly reaching levels in the 5000 range (Figure 1).

**Table 1:** Additional Lab Studies

Lab	Value	Reference Range
Hepatitis Bs Ag	Nonreactive	Nonreactive
Hepatitis Bs Ab	65.97 mIU/mL	>=12.00 mIU/mL
Hepatitis C Ab	Nonreactive	Nonreactive
HIV screen	Nonreactive	Nonreactive
C282Y HFE mutation	Negative	Negative
H63D HFE mutation	Negative	Negative
Copper level	85 mcg/dL	70-175 mcg/dL
Zinc level	57 mcg/dL	60-130 mcg/dL
Vitamin B1	171 nmol/L	78-185 nmol/L
Triglyceride	86 mg/dL	<149 mg/dL
Vitamin B12	792 pg/mL	180-914 pg/mL
Folate	35.1 ng/mL	>=5.9 ng/mL
TSH	1.948 uIU/mL	0.350-4.940 uIU/mL
ESR	23 mm/hr	0-22 mm/hr
CRP	0.16 mg/dL	0.00-0.74 mg/dL

**Figure 2:**
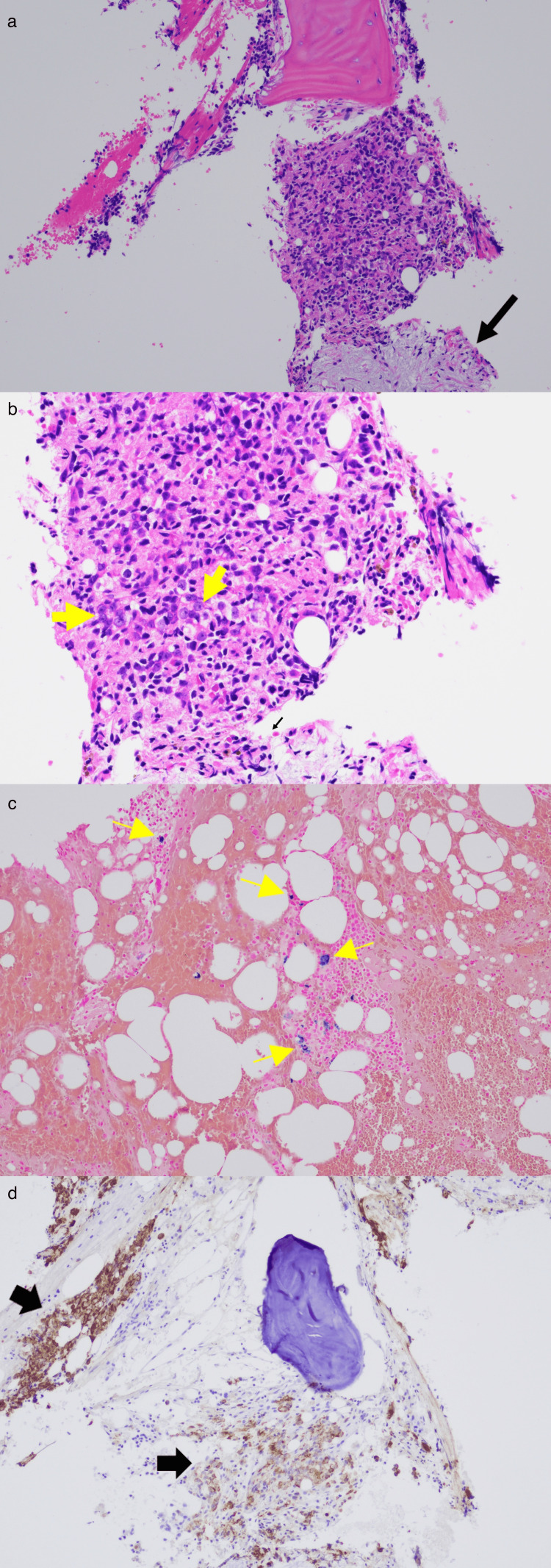
Bone Marrow Biopsy (a, b) Bone marrow biopsy showing sheets and clusters of large malignant cells (yellow arrows) and fibrosis (black arrow) (Hematoxylin and eosin stain, original magnification, × 100 (a), × 200 (b)). (c) Iron: Prussian blue stain performed on the bone marrow clot section shows adequate stainable iron stores. (d) The malignant cells are positive for pancytokeratin AE1/AE3, and negative for vimentin (not shown), supporting the diagnosis of metastatic carcinoma (immunohistochemical stain).

**Figure 3:**
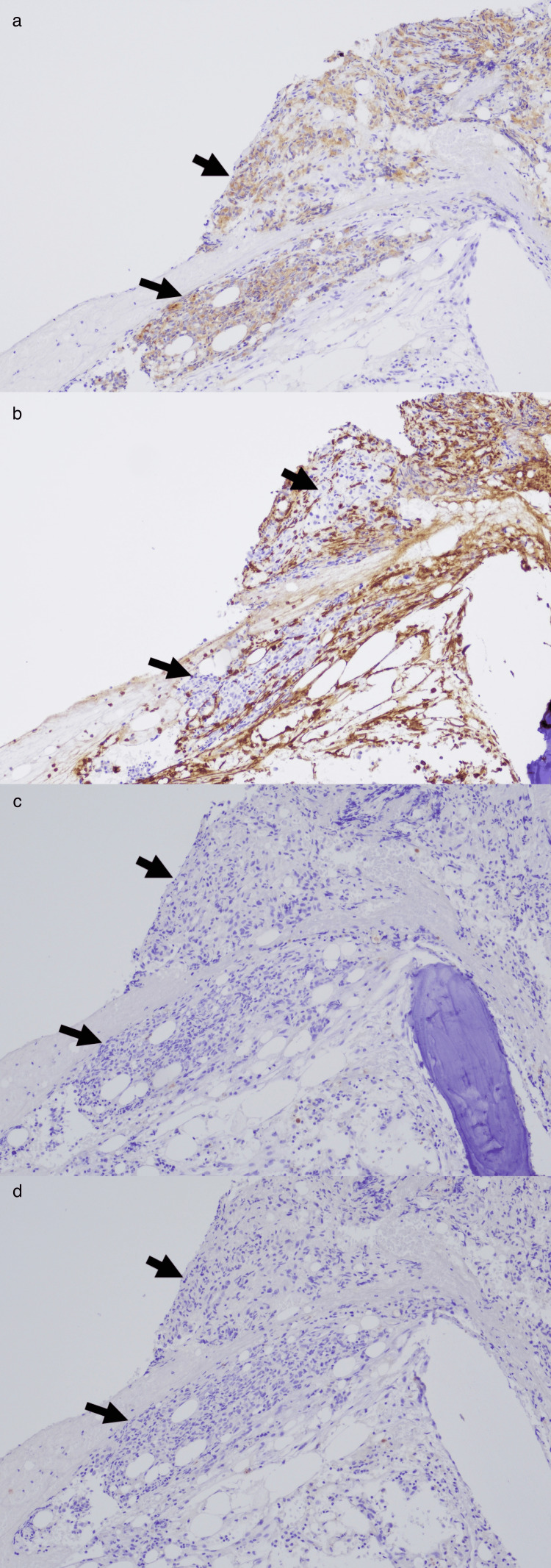
Further Immunohistochemical Stains Demonstrating Neuroendocrine Differentiation The tumor cells are weakly positive for synaptophysin (a), and negative for chromogranin (not shown), vimentin (b), CK7 (c), and CK20 (d).

By mid-2024, the patient ultimately agreed to a bone marrow biopsy for further evaluation of persistent hyperferritinemia and macrocytic anemia. Bone marrow aspirate and biopsy revealed metastatic carcinoma (Figures 2a, 2b) positive for pancytokeratin AE1/AE3 with adequate iron storage (Figure 2c), and negative for vimentin, CK7, and CK20 (Figure 2d). The bone marrow aspirate smears were largely hemodilute, showing predominantly peripheral blood with no bone marrow spicules. The bone marrow biopsy demonstrated a marked replacement of normal hematopoietic elements by sheets and clusters of malignant cells, accompanied by surrounding fibrosis (Figures 2a, 2b). Immunohistochemical stains revealed that the malignant cells were positive for pancytokeratin AE1/AE3, and negative for vimentin, CK7, and CK20 (Figure 2d). This immunohistochemical pattern can be seen in prostate adenocarcinoma, clear cell renal cell carcinoma, hepatocellular carcinoma, adrenal cortical carcinoma, small cell lung carcinoma, gastric adenocarcinoma, and others [12,13]. Additional stains showed tumor cells weakly positive for synaptophysin, negative for TTF-1, CDX-2, chromogranin, and inhibin but showed equivocal results for Hep-Parl, PSA, and PAX-8 (Figure 3). These findings suggested poorly differentiated metastatic carcinoma with neuroendocrine differentiation (NED) [12,13]. Given the bone marrow biopsy findings, computed tomography of the chest, abdomen, and pelvis did not identify any discrete or apparent mass. Other findings included a severely enlarged prostate and multiple foci of sclerosis involving the osseous structures of the pelvis. Given these findings, prostate cancer with NED was considered as an etiology. Further workup was recommended, but after extensive discussion, the patient and his family chose to defer further diagnostics, including prostate biopsy, PET/CT, and additional lab tests such as LDH, repeat PSA (4.4ng/mL in late 2017, 1.6ng/mL in mid-2022), and treatment with chemotherapeutic agents such as carboplatin and etoposide. The patient was subsequently referred for home hospice services and eventually passed away several weeks later.

## Discussion

Ferritin is a non-specific biomarker that can be elevated in iron storage disturbances, inflammation, autoimmune diseases, or malignancy. For this patient, the initial differential diagnosis was suspected to be iron overload from iatrogenic causes. However, in the months after the discontinuation of iron supplementation, iron, as well as ferritin levels, continued to worsen. Despite these lab abnormalities, the patient endorsed generalized fatigue that had been stable, without additional symptoms, and physical exam remained grossly benign. These findings prompted us to broaden the differential diagnoses (Table 2).

**Table 2:** Etiologies of Hyperferritinemia

Etiology	Specific Causes/Examples
Iron Overload Syndromes	Hereditary hemochromatosis, secondary iron overload (chronic transfusions, thalassemia, myelodysplastic syndrome, sideroblastic anemia, sickle cell disease, pyruvate kinase deficiency)
Infection	Acute and chronic infections (bacterial, viral (e.g., hepatitis, HIV, EBV, CMV), fungal, parasitic (malaria, dengue))
Chronic Liver Disease	Alcoholic liver disease, nonalcoholic fatty liver disease, chronic hepatitis B/C, acute hepatitis, cirrhosis, porphyria cutanea tarda
Renal Disease	Chronic kidney disease, nephrotic syndrome
Metabolic Syndrome	Obesity, diabetes mellitus, metabolic syndrome
Hereditary Hyperferritinemia without Iron Overload	Hereditary hyperferritinemia-cataract syndrome, ferroportin disease, aceruloplasminemia, atransferrinemia
Rheumatologic/Inflammatory	Adult-onset Still’s disease, systemic lupus erythematosus, rheumatoid arthritis, vasculitis, macrophage activation syndrome, hemophagocytic lymphohistiocytosis (HLH)
Malignancy	Hematologic malignancies (leukemia, lymphoma, myelodysplastic syndrome), solid organ malignancies
Others	Iatrogenic (parenteral iron, iron supplements), repeated blood transfusions, severe tissue injury, rare genetic/metabolic disorders

Primary iron storage disease, such as hereditary hemochromatosis, can directly lead to hyperferritinemia and was therefore considered first for this patient. However, tests for hemochromatosis mutation including C282Y and H63D were negative. Then, diseases of ineffective erythropoiesis and increased iron absorption leading to hyperferritinemia, such as sideroblastic anemia and thalassemia, were considered. However, these diseases were thought to be less likely, given low RBC count and macrocytosis. As an acute-phase reactant, hyperferritinemia can be observed in the setting of viral or bacterial infection. However, this patient lacked the signs, symptoms, and laboratory and imaging findings of infection, rendering them less likely. Due to immune-mediated ferritin stimulation by cytokines, autoimmune phenomena such as HLH were also considered. However, the patient lacked the constellation of findings associated with HLH. A diagnosis of HLH must meet five out of eight of the following criteria: fever, splenomegaly, cytopenias, hypertriglyceridemia, evidence of hemophagocytosis, low or absent natural killer cell activity, hyperferritinemia, and elevated soluble CD25 [14]. In a 2013 retrospective study of 627 patients with ferritin levels above 1,000 in an academic center, malignancy was most often diagnosed (153/627 patients) as the cause of the elevated ferritin. This was followed by iron overload syndromes (136 patients) [15]. Few diagnoses of HLH (six patients) and anemia of chronic inflammation (seven patients) were made, and in five patients, there was no clearly definable cause.

Hyperferritinemia can be observed in a variety of malignancies. A 2025 study screened an extensive dataset of 1.3 million patients with elevated ferritin values from the Israeli Maccabi Health Services, which showed a significant association between hyperferritinemia and a cancer diagnosis, with an odds ratio of 1.9 (95% confidence interval 1.71 - 2.15) [16]. In myelodysplastic syndrome (MDS), ineffective iron regulation may lead to hyperferritinemia. In a 2008 study of 2,994 patients with de novo MDS, a ferritin level of greater than 1,000 ng/mL served as a surrogate for iron overload and was strongly associated (hazard ratio (HR) 52.4, P<.0001) with decreased overall survival, as well as risk for acute myeloid leukemia (AML) transformation (HR 6.6, P<.0001) [17]. Similarly, hyperferritinemia can also be observed and serve as a prognostic factor in leukemias. In 2018, a Japanese multicenter retrospective study demonstrated that ferritin levels greater than 400 ng/mL had a negative event-free survival at 5 years (30% vs 40%; P=0.033) in AML patients [18]. In both AML and acute lymphoblastic leukemia (ALL), patients require frequent transfusions, and ferritin is used as a surrogate for iron overload. In a 2024 study of children with AML and ALL in one cancer center in Poland, 41% of children had serum ferritin (SF) over 500 ng/mL, and 14% had SF over 1000 ng/mL at the time of diagnosis. After treatment, 80% of children had SF over 500 ng/mL, and 31% had SF over 1000 ng/mL, with a positive correlation between SF and units of blood transfused [19]. In a 2021 prospective study of 29 breast cancer patients, when compared to a control of 59 healthy women, there was a positive correlation between ferritin levels and breast cancer (p = 0.083) [20]. A 2022 meta-analysis of 12 retrospective studies on patients (1654 total) with lung cancer showed that pre-treatment hyperferritinemia was associated with worse overall survival [21]. In GI malignancies, hyperferritinemia is observed in colorectal cancer [22,23], as well as hepatocellular carcinoma [24,25] patients. Across these GI studies, hyperferritinemia is shown to be associated with a worse prognosis. Lastly, hyperferritinemia is associated with disease-free survival and overall survival in a 2023 study of 367 patients with endometrial carcinoma [26].

Neuroendocrine neoplasms have distinct pathologic features. In poorly differentiated NEC, cytology may show a dirty smear with diffuse necrotic debris, cell polymorphism, severe nuclear fragility, salt and pepper chromatin, and frequent mitoses, often atypical [12]. Histology may show similar findings, including solid/organoid structure, abundant necrosis, abundant fibrous stroma, severe cell polymorphism, round-irregular shape, high nuclear/cytoplasm ratio, severe nuclear molding, salt and pepper chromatin, and frequent mitoses, often atypical. Immunohistochemistry may show CK +/- (dot-like), CgA +/-, Syn +, INSM1 +, SSTR2/5 -/+ hormones, high or very high Ki67, diffuse positive or global loss of p53, or Rb global loss. Among these markers, ferritin is relatively non-specific. Nevertheless, studies have shown that in pancreatic and lung neuroendocrine neoplasms, SF can be a valuable tumor marker for bone metastases [27,28].

In normal prostate tissue, there are three types of epithelial cells: basal, luminal, and neuroendocrine. In prostate cancer, these cells can transdifferentiate into neuroendocrine-like cells in a process called NED [29]. While the pathogenesis of NED remains poorly understood, synergistic physical interactions between the epithelial and neuroendocrine intraprostatic systems may be the main trigger [30]. Unfortunately, NED correlates with disease progression and poor prognosis [31,32]. As with the neoplasms discussed above, hyperferritinemia is positively associated with prostate cancer risk, with each 100 ng/mL increase in the SF level increasing the odds ratio of prostate cancer by 1.20 (95% CI: 1.13-1.36) in a case-control study published in 2017 [33].

In this case, bone marrow biopsy unexpectedly revealed poorly differentiated metastatic carcinoma with neuroendocrine differentiation. Typically, if malignancy were the cause for the patient’s elevated ferritin, we would have anticipated finding a primary hematologic neoplasm such as myelodysplastic syndrome or leukemia. There was little diagnostic clue that suggested neuroendocrine malignancy as the driving factor of the hyperferritinemia. The initial working diagnosis was metastatic prostate cancer that underwent neuroendocrine differentiation, as CT imaging revealed a severely enlarged prostate gland with sclerotic lesions at the pelvic bones without any other distinct masses. However, it is unclear whether the sclerotic bone lesions were from possible prostate cancer or another etiology completely. As the patient and family elected for hospice, further diagnostics such as prostate biopsy, PET/CT, and further lab testing such as LDH and PSA were unfortunately unable to be performed.

## Conclusions

Ferritin is the protein that serves as the main mechanism for iron storage, as well as an acute-phase reactant, rising in response to acute or chronic inflammation, infection, injury, autoimmune disease, or malignancy. This case highlights the non-specific nature of ferritin elevation and the resulting broad differential diagnoses that its elevation entails. When evaluating ferritin levels, it is important to consider both iron storage and potential underlying conditions that could cause hyperferritinemia. Malignancy should be considered as part of the differential, especially when other common etiologies of hyperferritinemia have been considered and ruled out. Specifically, this case highlights the subtle presentation of NECs in the setting of an asymptomatic patient with a benign hematologic picture. Only upon bone marrow biopsy with follow-up imaging did the NEC of prostate origin with sclerotic bone lesions emerge as the leading differential.
